# Association of sickle cell disease with anthropometric indices among under-five children: evidence from 2018 Nigeria Demographic and Health Survey

**DOI:** 10.1186/s12916-020-01879-1

**Published:** 2021-01-15

**Authors:** Mohammad Redwanul Islam, Md Moinuddin, Ayeda Ahmed, Syed Moshfiqur Rahman

**Affiliations:** 1 International Maternal and Child Health (IMCH), Department of Women’s and Children’s Health, Uppsala University, MTC-huset, Dag Hammarskjölds väg 14B, SE-75237 Uppsala, Sweden; 2 Institute of Child Health, University College London, 30 Guildford Street, London, WC1N 1EH UK; 3 Division of Maternal and Child Health, International Centre for Diarrhoeal Disease Research, Bangladesh (icddr,b), 68 Mohakhali, Dhaka, Bangladesh

**Keywords:** Sickle cell disease (SCD), Nutritional status, Anthropometric indices, Under-five children, Stunting, Hemoglobin, Mediation

## Abstract

**Background:**

Malnutrition continues to affect under-five children in Africa to an overwhelming proportion. The situation is further compounded by the burden of sickle cell disease (SCD). However, association of SCD with stunting, wasting, and underweight in a nationally representative sample of under-five children remains unexplored. We aimed to describe prevalence of undernutrition by sickle cell status, to evaluate its association with growth faltering ascertained by anthropometric indices, and to explore mediating role of hemoglobin.

**Methods:**

We availed data from the 2018 Nigeria Demographic and Health Survey (DHS) and the sample comprised 11,233 children aged 6–59 months who were successfully genotyped for SCD. The DHS employed a two-stage, stratified sampling strategy. SickleSCAN rapid diagnostic test was used for SCD genotyping. *Z*-scores of length/height-for-age (HAZ), weight-for-height (WHZ), and weight-for-age (WAZ) were computed against the 2006 World Health Organization Child Growth Standards. We fitted logistic regression models to evaluate association of SCD with stunting, wasting, and underweight. Mediation analysis was performed to capture the indirect effect of and proportion of total effect mediated through hemoglobin level in SCD-anthropometric indices association.

**Results:**

Prevalences of stunting, wasting, and underweight among children with SCD were 55.4% (54.5–56.4), 9.1% (8.6–9.7), and 38.9% (38.0–39.8), respectively. The odds of stunting were 2.39 times higher (adjusted odds ratio (aOR) 2.39, 95% CI: 1.26–4.54) among sickle children than those with normal hemoglobin. SCD was also significantly associated with underweight (aOR 2.64, 95% CI: 1.25–5.98), but not with wasting (aOR: 1.60, 95% CI 0.85–3.02). Association of SCD with all three anthropometric indices was significantly mediated through hemoglobin level: for SCD-HAZ, the adjusted indirect effect (aIE) was − 0.328 (95% CI: − 0.387, − 0.270); for SCD-WHZ, the aIE was − 0.080 (95% CI: − 0.114, − 0.050); and for SCD-WAZ, the aIE was − 0.245 (95% CI: − 0.291, − 0.200).

**Conclusion:**

We presented compelling evidence of the negative impact of SCD on anthropometric indices of nutritional status of under-five children. Integration of a nutrition-oriented approach into a definitive SCD care package and its nationwide implementation could bring promising results by mitigating the nutritional vulnerability of children with SCD.

## Background

Undernutrition continues to be entrenched in Africa. An estimated 149 million under-five children were stunted globally in 2018 and 39% of them lived in Africa. Wasting affected 49 million under-five children worldwide in 2018 and Africa was home to 28% of them. Africa is the only region that saw an escalation of the number of stunted children between 2000 and 2018 [1]. On the other hand, global burden of sickle cell disease (SCD)—the commonest inherited hemoglobinopathy—is disproportionately concentrated in sub-Saharan Africa [2]. Sickle cell anemia (SCA) is the most severe variant of SCD that occurs in those carrying two sickle cell genes. Sub-Saharan Africa hosts approximately 80% of the newborns with SCA globally. This proportion is projected to reach 88% by 2050. Nigeria accounts for 30% of the annual number of newborns with SCA, the highest country-level contribution to global tally [3]. Whereas the negative impact of SCD on children’s growth is well known [4], its association with conventional anthropometric indices in a nationally representative sample of Nigerian under-five children remains unexplored.

SCD and poor nutritional status of under-five children have several critical intersections. SCD mortality among African children is high: 50–90% of them reportedly die before 5 years of age [5]. Although lack of screening plays a role in this excessive mortality, many of the life-threatening complications of SCD among under-five children have well-recognized nutritional basis. Poor nutritional status is associated with impaired immune response to infections and, thus, drives infection-related mortality among children with SCD [4]. Nutritional status has also been implicated in the variability of SCD severity [6]. For instance, weight-for-age *z*-score (WAZ) has been shown to significantly predict hospitalization in SCD [7]. Therefore, nutritional status of under-five children is a promising avenue of intervention for reducing early SCD mortality and adverse prognosis [4]. Tackling undernutrition among under-five children with SCD will also contribute to overall progress toward Sustainable Development Goal Target 2.2 [8] in Nigeria—where high prevalences of growth faltering and SCD coincide. Characterization of SCD-nutritional status relationship at the national level is necessary to integrate nutritional approach to SCD management into a tailored public health package for children with SCD [9].

Children with SCD become anemic due to chronic hemolysis and low hemoglobin (Hb) level attenuates anthropometric parameters of growth [10, 11]. Moreover, cohort studies pinpoint Hb level as an independent risk factor for cerebral infarction in SCD [12]. More than one in five children with SCD suffers from overt or silent cerebral infarcts before turning 14 [13], yet stroke remains an under-recognized cause of early death among children with SCD in resource-limited settings. Accordingly, it is crucial to examine the mediating role of Hb level in SCD-growth faltering association. A substantial mediation will signal opportunity for dual benefit from interventions that improve Hb level: amelioration of growth and enhanced survival. Our objectives were to (i) describe and analyze prevalence of stunting, wasting, and underweight among under-five Nigerian children by sickle cell status and sociodemographic characteristics, (ii) evaluate association of SCD with growth faltering, and (iii) explore Hb level as a mediator in SCD-growth faltering association.

## Methods

### Study design, sampling, and participants

This cross-sectional study was based on secondary analysis of nationally representative data from the Nigeria Demographic and Health Survey 2018. It was conducted from August to December 2018 by the National Population Commission (NPC) in collaboration with the Federal Ministry of Health, along with technical assistance from The DHS Program, a United States Agency for International Development (USAID)-supported project [14].

**Fig. 1:**
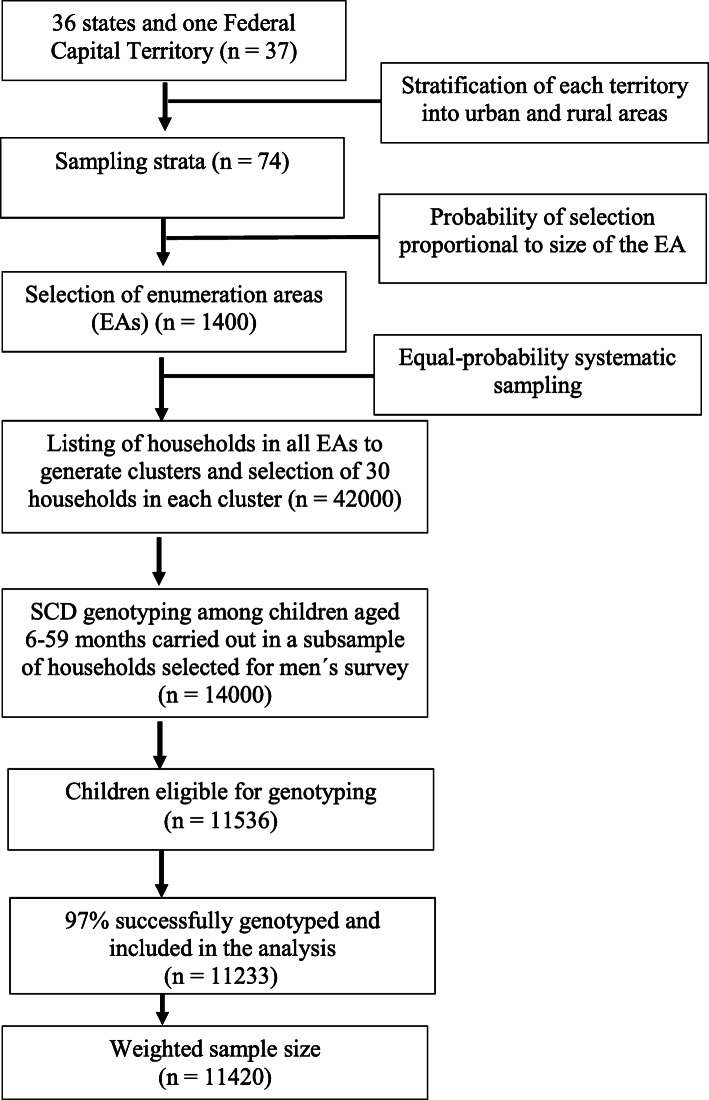
Flow diagram of children included in the analytic sample of this study

A two-stage stratified sampling strategy was adopted where 37 administrative territories were split into urban and rural areas, yielding 74 sampling strata. From these strata, 1400 enumeration areas (EAs) were selected. Thirty households from each EA were selected with equal-probability systematic sampling and in a third of those 42,000 households children aged 6–59 months underwent genotyping and anthropometric assessment [14]. The flow of participants into this study is illustrated in Fig. 1.

### Outcome variables

The outcome variables were three common anthropometric indices of nutritional status among under-five children: height-for-age, weight-for-height, and weight-for-age. These were standardized by constructing *z*-scores, using the World Health Organization 2006 Child Growth Standards [15]. The details of height and weight measurement can be found in the DHS final report [14]. Three forms of malnutrition were defined applying the WHO-recommended cutoffs. Stunting was defined as height-for-age *z*-score (HAZ) below − 2, wasting as weight-for-height *z*-score (WHZ) below − 2, and underweight as weight-for-age *z*-score (WAZ) below − 2. For further analysis, the three outcome variables were dichotomized: stunted versus non-stunted, wasted versus non-wasted, and underweight versus non-underweight, and coded binarily (“1” versus “0”).

### Explanatory variables

We used data from the Biomarker Questionnaire, Household Questionnaire, and Woman’s Questionnaire (DHS-7 version) to ascertain the explanatory variables selected according to existing knowledge mapped in a directed acyclic graph (DAG, Additional file 1: Fig. S1). The main variable of interest was sickle cell status of the children assessed with spot genotyping by SickleSCAN rapid diagnostic test kit [14]. Children with genotypes HbSS and HbSC were categorized as having SCD, those with genotypes HbAS and HbAC were categorized as “sickle cell and Hb C trait,” and those with genotype HbAA comprised non-sickle cell children. Children’s age in months was categorized into three groups (6–23, 24–40, and 41–59 months) according to tertiles of age distribution of the sample. Wealth index was derived from data on household ownership of a range of durables and such dwelling characteristics as source of drinking water, toilet facility, and flooring material using principal component analysis. It reflects socioeconomic status (SES). It was then divided into tertiles: the highest tertile representing the richest, intermediate tertile the middle-status, and the lowest tertile the poorest households in Nigeria. According to the highest level of education attended, maternal education was categorized into none, primary, and secondary or higher. We hypothesized place of residence to reflect access to health care, as urban under-five children are likely to have greater access than their rural peers. The DHS classified EAs with a population more than 20,000 as urban. Assessment of children’s Hb level in gram per deciliter (gm/dL) was performed on-site with HemoCue analyzer using capillary blood from finger/heel pricks [14]. Those with Hb < 11 g/dL were deemed anemic.

### Statistical analysis

All statistical analyses accounted for the two-stage stratified sampling strategy employed in DHS with “svyset” commands in Stata version 14.0 (Stata Statistical Software, College Station, TX, USA). Sampling weights were applied to compensate for the unequal probability of being recruited and to obtain nationally representative estimates. Distribution of continuous, numerical data was checked by examining quantile-quantile plots. HAZ, WHZ, WAZ, and Hb levels were approximately normally distributed. Weighted frequencies and proportions were computed for categorical data. Difference in prevalences of stunting, wasting, and underweight by sociodemographic characteristics was evaluated with chi-squared test, and difference in mean was tested with one-way ANOVA. Statistical tests were two-tailed and *P* values < 0.05 were considered statistically significant. We fitted binary logistic regression models to explore association of SCD with stunting, wasting, and underweight. Crude and adjusted odds ratios (OR) with 95% confidence intervals (CI) are reported. Collinearity between categorical, explanatory variables was assessed by cross-tabulation with chi-squared test. If found dependent (*P* < 0.05), Goodman–Kruskal’s gamma (G-K gamma) was computed to examine the strength of association. Maternal education (*P* < 0.05, G-K gamma = 0.76) and place of residence (*P* < 0.05, G-K gamma = 0.74) were strongly associated with SES and, therefore, were not included in the logistic regression. Furthermore, addition of these two variables in the adjusted model did not change effect estimates considerably (< 5%).

### Mediation analysis

**Fig. 2:**
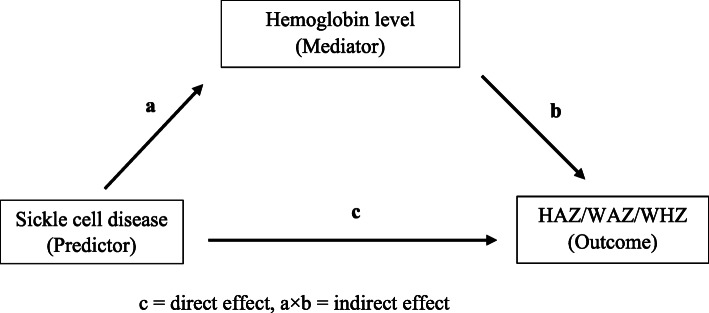
Path diagram showing Hb level as a mediator in SCD-anthropometric indices associations

For mediation analysis, we included children with sickle cell and Hb C traits (genotypes HbAS and HbAC) and those having normal hemoglobin (genotype HbAA) into one category versus children with SCD; converting the predictor into a dichotomous one. We tested three single-mediator models [16] for SCD-HAZ, SCD-WHZ, and SCD-WAZ associations with Hb level as a continuous, numerical mediator. Mediation analysis decomposes the “total” effect of exposure (SCD) on outcome (anthropometric indices) into “direct” and “indirect” effects. Figure 2 demonstrates the outline for mediation analysis: c represents the effect of SCD on the anthropometric indices after adjusting for the mediator (i.e., direct effect) and the effect of SCD on the indices through the mediator (i.e., indirect effect) is represented by a × b.

We retrieved standardized total, direct, and indirect effects, and proportion of the total effects mediated, both unadjusted and after adjusting for age, sex, and SES. Standard errors and CI were computed with bootstrapping (1000 repetitions). Mediation was assumed to occur if the indirect effect estimate, captured as average causal mediation effect (ACME), was statistically significantly different from zero. Following the recommendation by Hayes and Rockwood [17], we did not intend to identify the mediating role of Hb as complete or partial. A complete mediation by Hb level would be theoretically implausible owing to several other mechanisms that may mediate the SCD-anthropometric indices association [4], and we did not have data on those potential mediators. The mediated proportion was reported considering its public health relevance. Mediation analysis was also conducted in Stata.

### Ethics statement

The Nigeria DHS protocol was approved by the National Health Research Ethics Committee of Nigeria (NHREC) and the ICF Institutional Review Board. Enumerators obtained informed verbal consent prior to administering the questionnaires. Blood samples were collected from the children after acquiring consent from parents/guardians [14]. We obtained approval for accessing and using the data from The DHS Program.

## Results

Sample characteristics are presented in Table 1. The weighted analytic sample comprised 11,420 children aged 6–59 months and one third of them were younger than 24 months. The mean age of the sample was 32.2 months (standard deviation (SD) = 15.6). Slightly more than half of the children were male (51%). There were 8736 non-sickle children (HbAA), and 2352 children with sickle cell and Hb C trait (HbAS and HbAC). Genotypes HbSS and HbSC were detected among 102 and 34 children, respectively. The weighted prevalence of SCD was 1.3%. The majority of the households were relatively affluent (the Richest, 36.8%) and rural (56%). Nearly 46% of the children had mothers with secondary or higher education.

**Table 1:** Sociodemographic characteristics and weighted prevalences of stunting, wasting, and underweight by categories of sociodemographic variables and anemia (unweighted *n *= 11,233 and weighted *n* = 11,420)^a^

Characteristics	Weighted frequency^**b**^ (%)	Stunting prevalence (95% CI)	Wasting prevalence (95% CI)	Underweight prevalence (95% CI)
Age (in months):				
6–23	3916 (34.3)	33.4 (32.5–34.3)	11.9 (11.3–12.5)	23.0 (22.2–23.8)
24–40	3625 (31.7)	45.4 (44.5–46.3)	4.4 (4.0–4.8)	23.3 (22.5–24.1)
41–59	3879 (34.0)	38.0 (37.1–38.9)	3.7 (3.4–4.1)	20.5 (19.8–21.3)
		*P* < 0.001	*P* < 0.001	*P* = 0.017
Sex:				
Male	5827 (51.0)	41.5 (40.6–42.4)	8.1 (7.6–8.6)	23.8 (23.0–24.5)
Female	5561 (49.0)	35.9 (35.0–36.8)	5.4 (5.0–5.8)	20.7 (20.0–21.5)
		*P* < 0.001	*P* < 0.001	*P* < 0.001
Sickle cell status:				
Non-sickle cell (HbAA)	8829 (77.3)	38.2 (37.3–39.1)	6.6 (6.1–7.1)	21.8 (21.0–22.6)
Sickle cell and Hb C trait (HbAS, AC)	2440 (21.4)	40 (39.1–40.9)	7.2 (6.7–7.7)	22.9 (22.1–23.7)
Sickle cell disease (HbSS, SC)	151 (1.3)	55.4 (54.5–56.4)	9.1 (8.6–9.7)	38.9 (38.0–39.8)
		*P* = 0.004	*P* = 0.475	*P* = 0.006
Household SES:				
Richest	4197 (36.8)	22.4 (21.7–23.2)	4.3 (3.9–4.7)	11.5 (10.9–12.0)
Middle-status	3589 (31.4)	40.2 (39.3–41.1)	7.0 (6.5–7.5)	21.7 (20.9–22.4)
Poorest	3634 (31.8)	56.5 (55.5–57.4)	9.4 (8.8–9.9)	35.4 (34.5–36.3)
		*P* < 0.001	*P* < 0.001	*P* < 0.001
Place of residence:				
Urban	5030 (44.0)	28.3 (27.4–29.1)	5.2 (4.8–5.6)	15.4 (14.7–16.1)
Rural	6390 (56.0)	47.1 (46.2–48.0)	8.0 (7.5–8.5)	27.7 (26.8–28.5)
		*P* < 0.001	*P* < 0.001	*P* < 0.001
Maternal education:				
Secondary or higher	4743 (45.5)	22.1 (21.3–22.9)	5.0 (4.5–5.4)	12.0 (11.4–12.6)
Primary	1708 (16.4)	40.7 (39.8–41.7)	5.6 (5.2–6.0)	19.5 (18.7–20.3)
No education	3980 (38.1)	57.1 (56.2–58.1)	9.5 (8.9–10.0)	35.2 (34.3–36.1)
		*P* < 0.001	*P* < 0.001	*P* < 0.001
Anemic children (Hb < 11 g/dL):	7736 (67.9)	42.7 (41.8–43.7)	7.9 (7.4–8.4)	25.6 (24.8–26.4)
		*P* < 0.001	*P* < 0.001	*P* < 0.001

^a^All *P* values derived from chi-squared test. Missing data for variables: sex (*n* = 32), maternal education (*n* = 989), and anemia (*n* = 27)

^b^Weighted frequencies were rounded to the nearest integer

### Prevalences of stunting, wasting, and underweight by sickle cell status and sociodemographic characteristics

Overall prevalences of stunting, wasting, and underweight were 38.8% (37.9–39.7), 6.8% (6.3–7.2), and 22.2% (21.4–23.0), respectively. The prevalence of stunting increased from 33.4% among children younger than 24 months to a peak of 45.4% among children aged 24–40 months. Contrarily, the prevalence of wasting was highest among children below 24 months of age and declined thereafter. More than one in five children younger than 24 months were underweight. Prevalences of all three forms of malnutrition were higher among the male children. The highest sex difference of 5.6 percentage points was observed for stunting prevalence: 41.5% among male versus 35.9% among female children. Children with SCD had the highest prevalences of stunting (55.4%) and underweight (38.9%).

There was an ascending gradient of prevalence of all three forms of malnutrition from non-sickle cell (HbAA) children to those with SCD. Stunting was approximately 1.5 times more prevalent among children with SCD than their non-sickle cell peers (55.4% versus 38.2%). Underweight was roughly 1.8 times more prevalent among children with SCD compared with non-sickle cell children (38.9% versus 21.8%). The difference in wasting prevalence was statistically non-significant.

A socioeconomic gradient of ascending prevalence from more to less affluent households was also observed for all three forms of malnutrition. The prevalence of stunting among children from the poorest households was more than 2.5 times higher than that among children from the richest households (56.5% and 22.4%, respectively). Nearly one in 10 children from the poorest households were wasted. The prevalence of underweight was 23.9 percentage points higher among children from the poorest households than those from the richest households (35.4% versus 11.4%). Whereas 47.1% of rural children were stunted, 28.3% of their urban peers had stunting. Children of mothers with no education had a substantially higher prevalence of stunting (57.1%) than children born to mothers with higher educational attainment (22.1–40.7%). Wasting was more prevalent among children with uneducated mothers (9.5%) than children whose mothers had secondary education or above (5%). While the prevalence of underweight among children of mothers with secondary education or above was 12%, it rose to 35.2% among children with uneducated mothers. Approximately 43% of the anemic children were stunted and more than one in four anemic children were underweight. The prevalence of wasting among anemic children was 7.9% (Table 1).

### Mean HAZ, WHZ, and WAZ, and mean Hb level by sickle cell status

There was significant difference in all three mean *z*-scores when categorized by sickle cell status and children with SCD had the lowest means. Mean HAZ among children with SCD was 36.5% lower than non-sickle cell children (− 2.17 versus −1.59). Mean WAZ was 1.4 times lower among children with SCD in comparison to non-sickle cell children (− 1.63 versus − 1.13). While mean Hb level among children with SCD was more than 2 g/dL below than that of non-sickle cell children (8.04 g/dL versus 10.19 g/dL), mean Hb level of children with sickle cell trait was close to that of the latter (10.14 g/dL) (Table 2).

**Table 2:** Weighted mean HAZ, WHZ and WAZ scores, and weighted mean Hb level by sickle cell status among under-five children

Parameters	Non-sickle cell	Sickle cell and Hb C traits	Sickle cell disease	***P*** value*
Mean HAZ score^**a**^ (SD)	− 1.59 (1.57)	− 1.63 (1.58)	− 2.17 (1.47)	< 0.001
Mean WHZ score^**b**^ (SD)	− 0.30 (1.12)	− 0.36 (1.13)	− 0.52 (1.12)	< 0.001
Mean WAZ score^**c**^ (SD)	− 1.13 (1.24)	− 1.19 (1.23)	− 1.63 (1.16)	< 0.001
Mean Hb level in gm/dL^**d**^ (SD)	10.19 (1.56)	10.14 (1.40)	8.04 (1.65)	< 0.001

^a^Weighted *n* = 11,128

^b^Weighted *n* = 11,182

^c^Weighted *n* = 11,205

^d^Weighted *n* = 11,397

*P values derived from one-way ANOVA

### Association of SCD with stunting, wasting, and underweight

Table 3 demonstrates the results from multiple logistic regression analyses. When adjusted for age, sex, and SES, SCD was significantly associated with stunting and underweight, but not with wasting. The odds of stunting were 2.39 times higher among children with SCD when compared with non-sickle cell children (adjusted OR (aOR) 2.39, 95% CI 1.26–4.54). The odds of underweight were more than 2.5 times higher among children with SCD than their non-sickle cell peers (aOR 2.64, 95% CI 1.25–5.98). Magnitude of these associations increased in the adjusted model from the unadjusted one. Sickle cell and Hb C trait was not associated with stunting, wasting, or underweight.

**Table 3:** Logistic regression models analyzing association of SCD and sociodemographic variables with stunting, wasting, and underweight

	Stunting (HAZ < − 2 SD)		Wasting (WHZ < − 2 SD)		Underweight (WAZ < − 2 SD)	
	Unadjusted OR (95% CI)	Adjusted OR (95% CI)	Unadjusted OR (95% CI)	Adjusted OR (95% CI)	Unadjusted OR (95% CI)	Adjusted OR (95% CI)
Sickle cell status:						
Non-sickle cell (Hb AA)	1	1	1	1	1	1
Sickle cell and Hb C traits (Hb AS, AC)	1.09 (0.97–1.20)	1.07 (0.98–1.25)	1.01 (0.86–1.42)	1.09 (0.85–1.41)	1.06 (0.94–1.21)	1.07 (0.94–1.23)
Sickle cell disease (Hb SS, SC)	2.02 (1.24–3.27)	2.39 (1.26–4.54)	1.43 (0.74–2.76)	1.60 (0.85–3.02)	2.28 (1.26–4.14)	2.64 (1.25–5.98)
Age (in months):						
6–23	1	1	1	1	1	1
24–40	1.66 (1.48–1.86)	1.74 (1.53–1.98)	0.34 (0.27–0.43)	0.34 (0.27–0.43)	1.02 (0.90–1.16)	1.01 (0.88–1.16)
41–59	1.22 (1.10–1.37)	1.26 (1.12–1.41)	0.29 (0.23–0.36)	0.28 (0.22–0.36)	0.86 (0.76–0.97)	0.86 (0.76–0.97)
Sex:						
Male	1		1	1	1	1
Female	0.79 (0.72–0.86)	0.76 (0.70–0.83)	0.64 (0.54–0.79)	0.65 (0.55–0.78)	0.84 (0.76–83)	0.83 (0.75–0.92)
SES:						
Richest	1	1	1	1	1	1
Middle-status	2.32 (1.99–2.71)	2.36 (1.31–1.84)	1.68 (1.26–2.22)	1.71 (1.29–2.26)	2.14 (1.75–2.60)	2.16 (1.79–2.61)
Poorest	4.48 (3.85–5.22)	4.60 (3.95–5.36)	2.30 (1.77–2.97)	2.38 (1.84–3.07)	4.23 (3.50–5.12)	4.28 (3.57–5.13)

In comparison to those younger than 24 months, the odds of stunting were 74% higher among children aged 24–40 months (aOR 1.74, 95% CI 1.53–1.98). The odds of wasting were 72% lower (aOR 0.28, 95% CI 0.22–0.36) and that of underweight 14% (aOR 0.86, 95% CI 0.76–0.97) lower among those aged 41–59 months. Female sex was associated with lower odds of stunting (aOR 0.76, 95% CI 0.70–0.83), wasting (aOR 0.65, 95% CI 0.55–0.78), and underweight (aOR 0.83, 95% CI 0.75–0.92). SES showed an inverse association with the three forms of malnutrition. Children from the poorest households had more than fourfold higher odds of stunting compared with those from the richest households (aOR 4.60, 95% CI 3.95–5.36).

### Mediating role of Hb level

In the unadjusted analyses, we found all three SCD-anthropometric indices associations to be significantly mediated through Hb level. The statistically significant, negative indirect effects of Hb level became further pronounced in the adjusted analyses for all three associations. The adjusted average causal mediation effect (ACME) ranged from − 0.328 (95% CI − 0.387, − 0.270) for SCD-HAZ association to − 0.080 (95% CI − 0.114, − 0.050) for SCD-WHZ association. The proportion of total effect mediated through Hb level was highest for SCD-HAZ association (adjusted proportion mediated 0.928; 95% CI 0.535, 2.770), implying that the effect of SCD on HAZ was decomposed into two parts: nearly 93% of the total effect was indirect, i.e., mediated through Hb level and approximately 7% was direct effect (Table 4).

**Table 4:** Unweighted mediation analyses of Hb level as a mediator in the SCD-anthropometric indices association (*n* = 11,048)

	Unadjusted estimate	95% CI	Adjusted^**a**^ estimate	95% CI
**SCD-HAZ score association**				
Average indirect effect	− 0.444**	− 0.515, − 0.380	− 0.328**	− 0.387, − 0.270
Proportion mediated	1.358*	0.726, 4.800	0.928**	0.535, 2.770
**SCD-WHZ score association**				
Average indirect effect	− 0.134**	− 0.171, − 0.100	− 0.080**	− 0.114, − 0.050
Proportion mediated	0.609*	0.299, 2.900	0.373*	0.158, 2.010
**SCD-WAZ score association**				
Average indirect effect	− 0.342**	− 0.397, − 0.290	− 0.245**	− 0.291, − 0.200
Proportion mediated	0.982**	0.637, 2.190	0.664**	0.423, 1.320

^a^Adjusted for age, sex, and SES

**P < 0.01, *P < 0.05

## Discussion

We found significant variation in prevalences of stunting and underweight by sickle cell status, and these were markedly high among children with SCD. Prevalences varied significantly by sociodemographic characteristics as well. Stunting prevalence exceeded 50% among children from the poorest households. Mean *z*-scores of all three anthropometric indices were notably lower among children with SCD. Regression analyses revealed significant association of SCD with stunting and underweight. Hb level emerged as a statistically significant mediator in the association of SCD with all three anthropometric indices. The extent of mediation by Hb level was markedly high for SCD-HAZ association. To the best of our knowledge, this is one of the first analyses of association of SCD with anthropometric indices of nutritional status accounting for biological and socioeconomic correlates in a nationally representative sample.

The negative impact of SCD on anthropometric measures of nutritional status has been persistently captured in studies from Nigeria [18] and elsewhere [7, 19–24]. Proposed mechanisms fall into three broad categories: elevated resting energy expenditure (REE), reduced dietary intake, and metabolic and endocrine alterations [4, 25, 26]. A combination of increased protein turnover due to accelerated erythropoiesis and increased cardiac workload secondary to anemia and consequent hypoxia results in a higher REE among children with SCD [4]. Dietary intake is postulated to reduce from appetite suppression by high blood level of inflammatory mediators, particularly interleukin-6 [4], and during episodes of acute complications and hospitalization [27]. Dietary intake that is nutritionally adequate for children without SCD is unlikely to be sufficient for children with the condition because of the increased REE [4]. This may have driven growth faltering disproportionately among under-five children with SCD in Nigeria, where children’s dietary and feeding practices remain suboptimal and compounded by poverty and food insecurity [28, 29]. The extent to which suboptimal diet may amplify the impact of SCD on nutritional status in sub-Saharan Africa needs to be explored in future.

The magnitude of SCD’s association with stunting and underweight in our analyses was high with OR surpassing two. As the 2018 Nigeria DHS is the first DHS to implement SCD genotyping in a nationally representative sample [14], we could not find nationwide studies to compare the effect sizes with. In their prospective study involving a hospital-based cohort (*n* = 1618) in Tanzania, Cox et al. reported OR for stunting and underweight of 1.82 (95% CI 1.43–2.32) and 2.61 (95% CI 2.06–3.31), respectively, at enrolment. The mean age of the cohort was 10.1 years (range 0.5–48 years) [7]. Considering the older age distribution of participants, the lower OR for stunting versus our analyses (1.82 vs. 2.39) may reflect a survivor cohort effect [30]. The OR for stunting and underweight among children under 12 years of age were 4.87 (95% CI 2.94–8.06) and 4.74 (95% CI 2.96–7.60), respectively, in a cross-sectional study from the Democratic Republic of the Congo (DRC; *n* = 455) [21]. The higher OR compared to our analyses could be a manifestation of difference in HbS gene haplotype [31], as Bantu haplotype predominates in DRC, whereas Benin haplotype is pre-eminent in Nigeria [32].

Interestingly, we did not find a statistically significant association between SCD and wasting. This contradicts some of the previous studies [7, 23]. Pooled analyses show that the trajectory of mean WHZ in the first 5 years of life is different from that of mean HAZ and WAZ. Faltering in WHZ is concentrated between 3 and 15 months of age, and thereafter, it recovers steadily [33]. We posit that wasting could be an adaptive response to redirect energy and nutrients for maintaining essential metabolic processes [7], and it begins to accelerate as children with SCD live beyond the first 5 years. Of note, both studies [7, 23] finding significant SCD-wasting association had higher age distribution of participants with smaller number in the under-five category. Alternatively, the SCD-wasting non-association in this study may be a reflection of phenotypic difference owing to distinct HbS haplotypes [31].

The mean HAZ, WHZ, and WAZ of children with SCD in this study were significantly lower than their peers with HbAA genotype. The most severe deficit was observed for mean HAZ of children with SCD (− 2.17, SD 1.47). This is appreciably lower than mean HAZ from children with SCD in Ilesa, Nigeria (− 0.52, SD 1.33) [18]; Enugu, Nigeria (− 0.50, SD 1.57) [34]; Ghana (− 0.86, SD 1.40) [35]; and Kenya (− 1·10, SD 1·00) [36]. However, in the Kenyan SCD cohort (*n* = 128) with median age of 21.1 months, mean HAZ and WAZ did not significantly differ from those of non-sickle cell children [36]. No significant difference in mean HAZ was reported also in the study from Enugu, Nigeria [34] that recruited outpatient cases and controls from a tertiary hospital (*n* = 116). The mean age of cases in that study was 40.6 months (SD 16.79). The mean WAZ of − 1.63 (SD 1.16) among children with SCD found in our study is suggestive of a drastic left shift in weight-for-age trajectory compared with the WHO Child Growth Standards. An unitary increase in WAZ reduces hospitalization risk among children with SCD by 13% [7]. Besides, mortality risk for sickle children with WAZ below − 3 can be threefold higher (adjusted hazard ratio 3.42; 95% CI 2.50–4.68) [37]. The low mean WAZ in our study, therefore, signals increased likelihood of adverse prognosis for community-dwelling Nigerian children with SCD lacking definitive care. Moreover, the better anthropometric indices recorded among facility-recruited children with SCD indicates that early diagnosis and access to care can minimize growth faltering to a large extent.

The mean Hb level of sickle children in our study (8.04 g/dL, SD 1.65) was similar to that found in a multi-country (Mali, Senegal, Cameroon, Gabon, and the Ivory Coast) analysis [38], and in studies from Ghana [35] and Brazil [23], but higher than that of Kenyan cohorts [36, 39]. We captured a significant mediating role of Hb in SCD-anthropometric indices association. While this is unsurprising as chronic hemolytic anemia lies at the core of pathophysiologic alterations in SCD [31], the extent of mediation has enormous public health implication. Robust studies show Hb concentration to positively correlate with *z*-scores of height, weight, and BMI as well as growth velocity in SCD [10, 20]. Our mediation analysis corroborates this by pinpointing the extent of SCD’s impact on conventional anthropometric indices mediated through Hb level. Approximately 93% of the effect of SCD on HAZ, 37% of that on WHZ, and 66% of that on WAZ was mediated through Hb level. Nonetheless, these estimates merit careful interpretation as we could not include all potential mediators in our analysis and the data came from a cross-sectional survey. Furthermore, low Hb is a consistent predictor of stroke and mortality among children with SCD [12, 40]. Interventions to optimize Hb level among under-five children with SCD including universal point-of-care screening in early infancy, hydroxyurea therapy, and judicious use of blood transfusion [9] are of relevance in this regard. The pitfalls of blood transfusion in African settings concern unavailability of transfusable blood, transmission of infections, and alloimmunization. Local trials are needed to guide context-appropriate use of blood transfusion for under-five children with SCD in Nigeria and to examine its impact on nutritional status and mortality [40].

Some limitations of this study must be acknowledged for allowing careful interpretation of the findings. Causal inferences cannot be drawn from the findings of mediation analysis because of the lack of temporality in association. It is noteworthy that although clinical presentation takes time to fully develop, the genetic changes underlying SCD are present at birth. The rapid diagnostic test (SickleSCAN) used for SCD genotyping in the DHS showed diagnostic sensitivity and specificity of 85% and 98%, respectively, when compared with high-performance liquid chromatography [14]. We did not have data on energy intake, blood nutrient levels and HbF concentration, which could have made the analysis comprehensive. We adjusted for age, sex, and SES in the statistical model, but residual confounding could not be ruled out. Generalizability of the results to other countries in sub-Saharan Africa could be limited by phenotypic variation in SCD and difference in sociodemographic attributes.

## Conclusion

We presented compelling evidence of the association of SCD with stunting and underweight among under-five children in a setting burdened with childhood undernutrition and an under-five mortality rate of 132 per 1000 live births [14]. Mediation analysis indicated that measures to improve Hb level of Nigerian children with SCD may safeguard against growth faltering and enhance survival. Deteriorating nutritional status poses considerable threat to well-being of children with SCD and renders them prone to adverse prognosis. Educating parents on nutritional requirements in SCD and targeted supplementation with specific micro- and macronutrients may prove useful. Integration of such a nutrition-oriented approach into a definitive SCD care package [9] and its implementation at scale is needed to mitigate nutritional vulnerability of these children.

## Supplementary Information

**Additional file 1: Figure S1.** Directed acyclic graph (DAG) for the association of sickle cell disease with anthropometric indices. Lines in green are potential causal paths and those colored red indicate potential biasing paths that need to be adjusted in the model.

## Data Availability

Datasets are available upon request from The DHS Program website: https://dhsprogram.com/data/dataset/Nigeria_Standard-DHS_2018.cfm?flag=0. The datasets analyzed during the current study are available on reasonable request.

## Abbreviations

SCD: Sickle cell disease
DHS: Demographic and Health Survey
HAZ: Height-for-age *Z*-score
WHZ: Weight-for-height *Z*-score
WAZ: Weight-for-age *Z*-score
aOR: Adjusted odds ratio
aIE: Adjusted indirect effect
Hb: Hemoglobin
NPC: National Population Commission, Nigeria
USAID: United States Agency for International Development
EA: Enumeration area
DAG: Directed acyclic graph
SES: Socioeconomic status
CI: Confidence interval
G-K Gamma: Goodman-Kruskal’s gamma
ACME: Average causal mediation effect
NHREC: National Health Research Ethics Committee, Nigeria
ICF: Inner City Fund
SD: Standard deviation
REE: Resting energy expenditure
DRC: The Democratic Republic of The Congo
HbF: Hemoglobin F (fetal hemoglobin)
