# Supplementary figures and images for: Association of sickle cell disease with anthropometric indices among under-five children: evidence from 2018 Nigeria Demographic and Health Survey
Source: BMC Med. 2021 Jan 15;19:5. doi: 10.1186/s12916-020-01879-1 (PMC7809862; doi:10.1186/s12916-020-01879-1)

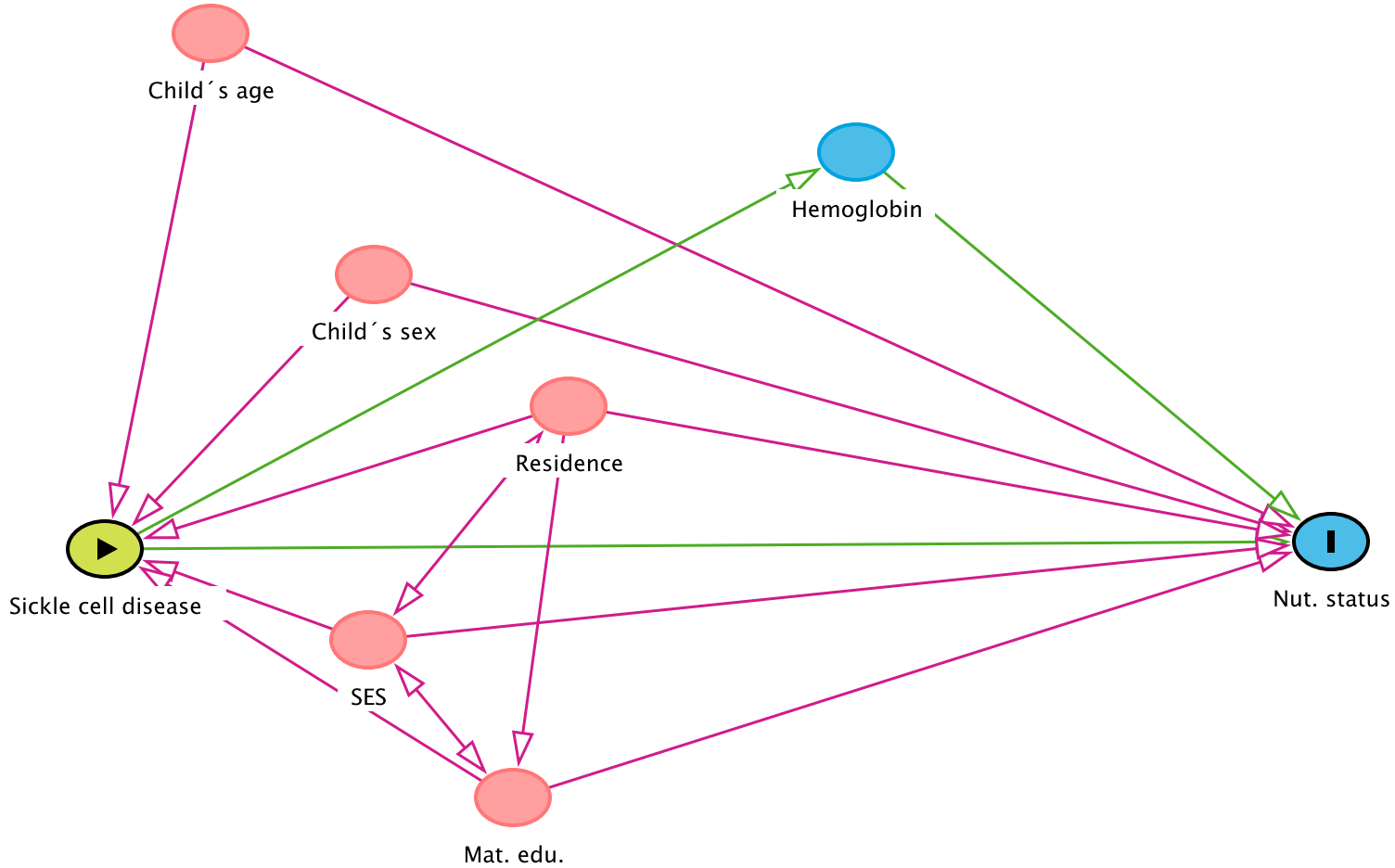

Supplement: Supplementary file 1 — Additional file 1: Figure S1. Directed acyclic graph (DAG) for the association of sickle cell disease with anthropometric indices. Lines in green are potential causal paths and those colored red indicate potential biasing paths that need to be adjusted in the model. [file 12916_2020_1879_MOESM1_ESM.pdf]
